# Regulation of GSK3β-FBXW7-JUNB Axis

**DOI:** 10.18632/oncotarget.1151

**Published:** 2013-07-04

**Authors:** Beatriz Pérez-Benavente, Rosa Farràs

**Affiliations:** Departamento de Bioquímica y Biología Molecular, Facultad de Medicina y Odontología, Universidad de Valencia, Valencia, Spain; Oncogenic Signalling Laboratory, Centro de Investigación Príncipe Felipe, Calle de Eduardo Primo Yúfera, 5, 46012 Valencia, Spain

In the last decade the involvement of the activator protein 1 (AP-1) in human cancer has been demonstrated. AP-1 proteins can be oncogenic on their own in certain situations; however, the major contribution of AP-1 to tumorigenesis is as a downstream effect of oncoprotein signaling [1]. The AP-1 transcription factor is a homo- or heterodimer combination of bZIP proteins belonging to the JUN, FOS, ATF and MAF families. In response to mitogenic and stress signals AP-1 binds to specific DNA regulatory elements of its target genes and transactivates or represses them. It is involved in the regulation of many basic cell processes, such as apoptosis, differentiation, proliferation, stress response and inflammation. We have recently reported in a paper published in Oncogene [2] novel molecular events that coordinate the degradation of JUNB, a member of the Jun family, in G2 and described the pathological consequences of its dysregulation through NPM-ALK signals in Anaplastic large cell lymphoma (ALCL). The *JUNB* gene was originally discovered as an immediate early growth response gene in mammalian cells. Early studies suggested that JUNB was an inhibitor of cell proliferation and transformation, antagonizing c-Jun activity. Later, JUNB was shown to have both cell cycle inhibitory and proliferation promoting activities depending on the cell context [3]. The inhibition of cell cycle and tumor suppression action of JUNB can be explained by its capacity to induce transcription of the cyclin-dependent kinase inhibitor *p16INK4α* and the repression of *cyclin D1*. Clinical observations have also established the activity of JUNB as a tumor suppressor in chronic (CML) and acute myeloid leukemia (AML). Moreover, JUNB negatively regulates the proliferation of hematopoietic stem cells suggesting that the oncogenic process leading to myeloid tumors starts in these cells [4]. On the other hand, oncogenic actions for JUNB have been described. In this regard, JUNB has been shown to cooperate with c-Jun in the development of mouse fibrosarcoma, and to promote cell proliferation by activation of *cyclin A* transcription. Furthermore, it has been shown to contribute to lymphoma pathogenesis in humans [3]. JUNB protein levels are tightly regulated during the cell cycle [5]. Similarly to c-Jun, JUNB levels are very low in quiescent cells. Its expression is rapidly and transiently induced by mitogenic stimuli during the G0/G1 transition before it returns to an intermediate level, both events being instrumental for progression towards S phase. Then, JUNB abundance decreases in mid/late G2. In contrast, c-Jun levels do not vary during the cell cycle. As a difference from JUNB, c-Jun is phosphorylated on its N-terminal serines by the JNK increasing its transactivational potential and stability from G2 to M [6]. Low JUNB levels in mitosis allow c-Jun to induce *cyclin D1* transcription and progression into G1. Therefore, member-specific Jun modifications seem to be important for the regulation of their protein expression during cell cycle progression. We have previously reported the abrupt disappearance of JUNB by mid-G2 as an essential step for proper mitosis [5]. In our published article [2] we provide evidence of the molecular mechanism involved in JUNB degradation in G2. We have found that GSK3β-mediated phosphorylation of JUNB on a critical consensus phosphodegron induces FBXW7 E3-ligase recruitment and its degradation in late G2. GSK3-mediated phosphorylation requires a priming phosphorylation by a still unknown kinase at the +4 position (serine 259 in JUNB). We also reported that abnormal conditions that stabilize JUNB, including mutations in the consensus phosphodegron or deletion of the *FBXW7*, lead to transcriptional activation of *cyclin A2* and repression of *DDX11*, a DNA helicase essential for sister chromatid cohesion. Consequently, JUNB stabilization conveys sister chromatid cohesion defects associated with mitotic catastrophe. Dysregulation of GSK3β-FBXW7-JUNB axis may be relevant in cancer, since we demonstrated that the mechanism regulating JUNB destruction in G2 is altered in ALK-positive ALCL, thus causing mitotic aberrations.

**Figure 1:**
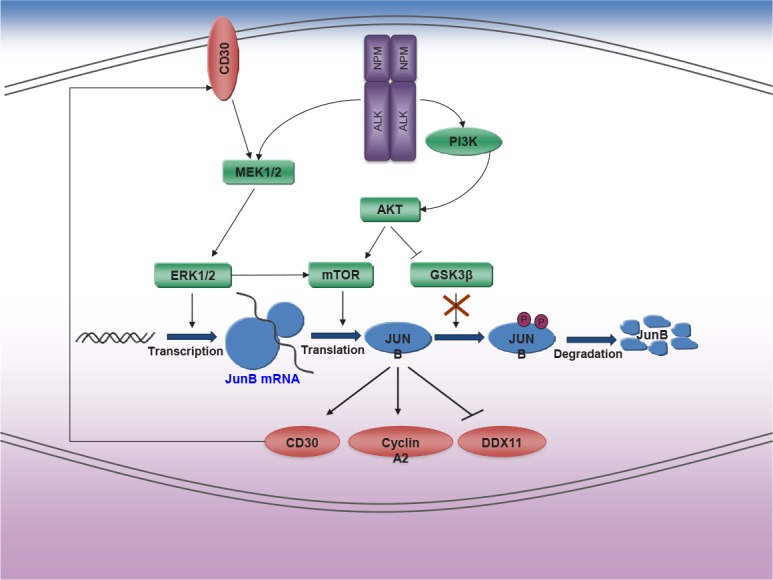
Expression of JunB in ALK+ ALCL JUNB is the main AP-1 transcription factor involved in the pathogenesis of ALCL. Three mechanisms for abnormal JUNB accumulation have been described in ALK-positive ALCL, (i) increased *JUNB* transcription dependent on Erk1/2 kinase activation by NPM-ALK, (ii) increased *JUNB* translation mediated by mTOR activation by AKT, which is induced by NPM-ALK via activation of PI3K, and (iii) impaired JUNB degradation by a substantial decrease in GSK3β activity through phosphorylation at S9 by constitutive activation of PI3K/AKT.

ALCL is an aggressive type of non-Hodgkin lymphoma of the T-cell/null lineage frequently associated with chromosomal translocations involving the anaplastic lymphoma kinase (*ALK*) locus on chromosome 2. The most common translocation results in the expression of the NPM-ALK fusion oncogenic kinase. JUNB is overexpressed in ALK-positive ALCLs [7]. Its abnormally high accumulation is explained by increased *JUNB* transcription and translation by NPM-ALK (Figure 1). We have uncovered a novel mechanism that regulates JUNB protein levels through NPM-ALK signaling. Thus, constitutive oncogenic activation of ALK inhibits GSK3β activity via activation of PI3K. As a result degradation of JUNB protein is impaired, cyclin A2 is up-regulated and DDX11 down-regulated in mitosis, resulting in mitotic aberrations, including premature sister chromatid separation in metaphase. In addition, inhibition of GSK3β activity by NPM-ALK may lead to stabilization of numerous proteins whose degradation is dependent on its kinase activity. In fact, McDonnell et al. [8] showed that inactivation of GSK3β resulted in the accumulation of CDC25A and MCL1, which confer the advantage of growth and protection from apoptosis in ALK+ ALCL.

These findings provide new molecular insights on JUNB-dependent neoplastic transformation. It will be of interest to determine in depth JUNB dependent gene expression and dimerization partners under pathological conditions in order to antagonize or inhibit its oncogenic activity, in combination with PI3K/AKT and/or NPM-ALK pathway blockade.
